# Legionnaires’ disease outbreak linked to a cold-water source in Yorkshire, 2022

**DOI:** 10.1017/S0950268825100113

**Published:** 2025-05-30

**Authors:** Alexander Thompson, Aston Quinney, Rob Johnston, Lauren Margrove, Jane Bradbury, Phil Roberts, Baharak Afshar, Gareth J. Hughes, Mercy Vergis

**Affiliations:** 1 Yorkshire and Humber Health Protection Team, UK Health Security Agency, Leeds, UK; 2 North East and Yorkshire and Humber Field Services, UK Health Security Agency, Leeds, UK; 3 Food, Water and Environmental Microbiology Laboratories, UK Health Security Agency, York, UK; 4 Local Authority Environmental Health; 5 Health Unit, Health and Safety Executive, Bootle, UK; 6 Zoonotic and Acute Respiratory Section, Respiratory and Vaccine Preventable Bacteria Reference Unit, UK Health Security Agency, London, UK

**Keywords:** Legionnaires’ disease, *Legionella*, cold-water system, Environmental health, enforcement, heat transfer

## Abstract

We describe an outbreak of Legionnaires’ disease linked to an exclusive cold-water source in a private residential setting in Yorkshire. The cold-water source was identified following microbiological testing of clinical and environmental samples. *Legionella pneumophila* was only detected in the cold-water system. Three cases were identified over the course of the outbreak: two confirmed and one probable. Conditions favourable to bacterial growth included system ‘dead legs’ and significant heat transfer to the cold-water system. We describe challenges in implementing control measures at the venue and highlight the importance of using enforcement powers, where necessary, to reduce risk.

## Summary page

We describe an outbreak of three cases of Legionnaires’ disease (LD) linked to an exclusive cold-water source in a private residential setting in Yorkshire. The purpose of this outbreak report is to highlight the potential risk to public health from inadequately designed or maintained domestic cold-water systems and to describe learning from our response to the outbreak. The report describes epidemiological and microbiological findings and the control measures implemented by the Incident Management Team. We found three LD cases that were linked epidemiologically, and the sputum sample of one case was a partial match for the *Legionella pneumophila* cultured from environmental samples. We found conditions favourable to bacterial growth at the setting, including system ‘dead legs’ and significant heat transfer to the cold-water system. We describe challenges in engaging the private accommodation provider, although engagement improved significantly following enforcement action, in relation to the Health and Safety at Work, etc. Act 1974, taken by the relevant enforcing agency, which, in this case, was the Health and Safety Executive. If breaches of health and safety legislation are suspected, then we advise early engagement with the relevant enforcing agency to establish any potential role within their legal remit.

## Introduction

Legionnaires’ disease (LD) is an atypical form of pneumonia caused by species of the Gram-negative bacteria genus *Legionella*, of which *Legionella pneumophila* Serogroup 1 is the primary causative agent for LD. Symptoms range from mild disease to hospitalization and death [1]. Older patients, and individuals with underlying medical conditions, such as chronic lung disease or immunosuppression, are known to be at higher risk [2].

LD is a source of preventable ill-health and death. In 2023, there were 604 cases of LD notified in England and Wales, giving an incidence rate of 1/100 000 population in England, and 1.4/100 000 population in Wales [3]. While over a third of cases in 2023 were associated with foreign travel (35.1%), most cases of LD occur in the community (62.7%) [3]. The World Health Organization reports a typical LD case fatality rate of between 5% and 10% [4], although the most recent case fatality rate in England and Wales was reported to be lower (~3%) [3].

LD can be transmitted via the inhalation of infectious aerosols from a colonized source. Onward person-to-person transmission does not occur [1]. *Legionella* species are ubiquitous in aquatic environments and multiply readily within the temperature range of 25°C–45°C [5]. Consequently, advice for preventing *Legionella* colonization in a water distribution system typically involves distributing hot water at 50°C or higher, while cold water should be stored and distributed below 20°C [5]. *Legionella* colonization of cold-water systems has been recognized in the literature [6]. However, outbreaks of LD are far more commonly linked to hot-water distribution systems [7, 8]. The authors are aware of two published outbreak reports, both in healthcare settings, in which the identified source of *Legionella* was exclusively the cold-water system [9, 10]. To the best of our knowledge, this is the first published outbreak report of LD linked to an exclusive cold-water source in a domestic residential setting.

The control of *Legionella* in a private domestic setting poses unique challenges. While the Health and Safety Executive (HSE) provides guidance and a Code of Practice on the control of *Legionella* in these settings [11], compliance may be difficult to monitor and enforce, while implementing sufficient control measures may come at significant cost to the private provider. Local authorities have a key role in evaluating compliance with these standards. Where compliance falls below statutory requirements, enforcing authorities can use statutory powers to require mitigation works.

Here, we describe an outbreak of LD in a private residential setting in Yorkshire. The outbreak was atypical in that the most likely source was exclusively the cold-water system within the residence. We highlight challenges arising in the management of the outbreak in this setting, and the importance of collaborative working with colleagues across the public health system, including the UK Health Security Agency (UKHSA), Environmental Health in the Local Authority, and HSE.

## Outbreak description

The first case of urinary antigen-positive LD was notified to UKHSA’s regional Health Protection Team on 7 October 2022. Case details were gathered using a national surveillance questionnaire [12]. The questionnaire considers different risk factors for *Legionella* exposure, including spas, hot tubs, and air conditioning units. No obvious risk factors for exposure emerged from this history-taking, and while the patient had recently moved into a new accommodation, this was not considered a potential source of infection since clinical symptoms had begun prior to the move. No further investigation of the accommodation occurred at this stage.

The second case of urinary antigen and Polymerase Chain Reaction (PCR)-positive LD was notified on 3 November 2022. It was identified that this second case was also a resident at the same accommodation, and therefore further enquiries about the venue were initiated, including an on-site assessment from Environmental Health Officers (EHOs) of the local authority. An Incident Management Team (IMT) was convened, including attendees from UKHSA, Environmental Health, Local Authority Public Health, the National Health Service, and HSE.

Thirty-four environmental samples were taken at the venue. Sample sites included the flats of the two cases, three further flats chosen at random, and the communal shower rooms. Samples considered to be unsatisfactory for *Legionella* were those with a test result >1 000 colony-forming unit (CFU)/L. Samples from the cold water taps and showers of the two flats of the identified cases had around 30 000 CFU/L. Only the cold-water samples produced unsatisfactory results. While multiple samples were taken from hot-water outlets, none of these were unsatisfactory (all <20 CFU/L).

Urine and a lower respiratory sample (sputum) from Case 2 were tested at the Respiratory and Vaccine Preventable Bacteria Reference Unit, UKHSA. *L. pneumophila* antigen was detected in the urine sample, and *L. pneumophila* Serogroup 1 DNA was detected in sputum using an in-house qPCR assay. Unfortunately, *L. pneumophila* was not isolated from the sputum sample; however, direct nested sequence-based typing (SBT) yielded a partial allelic profile (for four out of seven alleles) (0, 0, 0, 3, 14, 9, 11) consistent with sequence types (STs) 9, 107, 347, 579, 1315, 1801, 2066, and 2925, or a new or mixed ST. A total of seven environmental isolates were identified as *L. pneumophila* Serogroup 1, ST 9 using the ‘gold-standard’ SBT method. The partial allelic profile from the clinical sample (0, 0, 0, 3, 14, 9, 11) was consistent with ST 9 (3, 10, 1, 3, 14, 9, 11) from the environmental isolates from the premises (Table 1). Given the epidemiological and (partial) microbiological link between the accommodation and the cases, the absence of other plausible exposures in the case histories, and the unsatisfactory results exclusively from the cold-water system samples, the IMT agreed that the cold-water system at the residence was the most likely source of the outbreak.

**Table 1.** Microbiological test results

Samples	Urinary antigen tests (urine)	PCR (sputum)	Culture (sputum)	Typing – allelic profile	ST
Case 2	*Legionella pneumophila* detected	*L. pneumophila* sg1 detected	*Legionella* species not isolated	0, 0, 0, 3, 14, 9, 11^a^	Partial allelic profile consistent with different STs, including ST 9
Environmental isolate 1			*L. pneumophila* sg1 isolated	3, 10, 1, 3, 14, 9, 11	ST 9
Environmental isolate 2			*L. pneumophila* sg1 isolated	3, 10, 1, 3, 14, 9, 11	ST 9
Environmental isolate 3			*L. pneumophila* sg1 isolated	3, 10, 1, 3, 14, 9, 11	ST 9
Environmental isolate 4			*L. pneumophila* sg1 isolated	3, 10, 1, 3, 14, 9, 11	ST 9
Environmental isolate 5			*L. pneumophila* sg1 isolated	3, 10, 1, 3, 14, 9, 11	ST 9

Case 2: *L. pneumophila* urinary antigen was detected using Binax and Bartels enzyme immunoassays (EIA). *L. pneumophila* Serogroup 1 was detected by PCR in sputum. *Legionella* spp. was not isolated. The partial allelic profile from Case 2 was consistent with the environmental isolates’ ST (ST 9). The urine sample from Case 1 was not sent to the reference laboratory, and no sputum sample was taken. ST, sequence type.

a Direct nested sequence-based typing method yielded a partial allelic profile (0, 0, 0, 3, 14, 9, 11) consistent with STs 9, 107, 347, 579, 1315, 1801, 2066, and 2925, or a new ST or mixed STs.

A further probable case was identified in November 2022 following interviews between EHOs and residents at the venue. This probable case had been hospitalized with respiratory symptoms earlier in October. Unfortunately, no microbiological testing for *Legionella* occurred at the time of admission, and therefore it was not possible to formally confirm an LD diagnosis.

**Figure 1.**
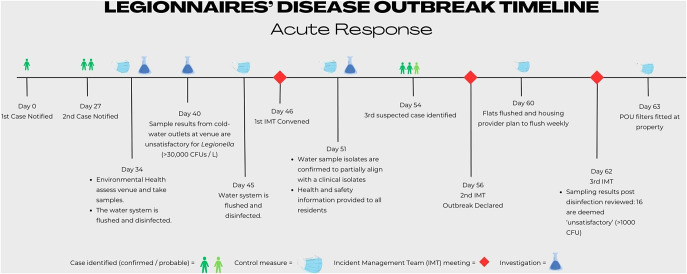
Timeline of the acute response to the Legionnaires’ disease outbreak.

A second formal meeting of the IMT was convened on 2 December 2022. At this meeting, an LD outbreak was declared. Case definitions were agreed, and the national LD case definition, incorporating microbiological, clinical, and radiological findings, was amended to include an epidemiological link to the identified venue after 1 October 2022 [13]. Two of the identified cases were ‘confirmed’ since there was microbiological evidence of *Legionella* infection and an epidemiological link to the venue. One case remained ‘probable’ since this patient had no biological specimens tested for *Legionella* during their illness, although clinical and radiological findings supported an LD diagnosis. All three patients were adults over the age of 60. A timeline of the acute response to the outbreak is presented in Figure 1.

## The context

The setting for the outbreak was a privately run ‘sheltered’ accommodation building. ‘Sheltered’ accommodation generally describes accommodation for elderly or disabled people and incorporates individual dwellings in addition to some shared facilities and support from a warden. In this case, many of the residents were considered vulnerable due to underlying medical conditions.

The building contained 36 flats (maximum occupancy of two per unit), each with its own kitchen and bathroom. The water system was run communally throughout the whole estate. Cold water was supplied directly from the mains water supply, while hot water was stored and supplied from the communal ground-floor boiler room. While the temperature of the water could be isolated and controlled in each flat, the temperature of the water that came into the flats was controlled by the whole system. In the flats, cold and hot tap water was accessed via separate outlets; in the communal areas, thermostatic mixing valves were present for mixer taps. Showers were electric and had mixing valves.

Over the course of the outbreak investigation, two important structural conditions favourable to the growth of *Legionella* were identified: system ‘dead legs’ and inadequate pipe insulation in the context of raised environmental temperatures. ‘Dead legs’ are sections of a water system’s pipework in which the water can become stagnant due to little or no flow. Three ‘dead legs’ were identified at the venue. In addition to the risk from stagnant water, we found that the environmental temperature at the venue was high enough to warm the cold-water piping system, which was not insulated as is typical of indoor plumbing in the United Kingdom.

At the accommodation, the radiators in the hallways and communal areas were set centrally to a high temperature and were always on, even overnight, preventing the fabric of the building from cooling down. Consequently, the uninsulated pipework allowed heat transfer to occur due to persistently high ambient air temperatures. Temperature samples from the cold-water system, taken after a 2-minute flush, were typically around 25°C–29°C, with the highest measured at 32°C. Since none of the microbiological samples from hot-water outlets had unsatisfactory results, no temperature sampling from the hot-water system was undertaken.

## Control measures

Control measures instigated over the course of the outbreak can be summarized under three main aims: preventing transmission of *Legionella*, decolonizing the water system, and removing the conditions favourable to bacterial growth. To prevent transmission, point-of-use filters were fitted to all taps and shower heads, in both the flats and communal areas, 1 week after the outbreak was declared. Point-of-use filters are commonly used in outbreaks to prevent *Legionella* exposure where a water system is contaminated [14]. While the IMT had considered rehousing residents at this stage, it was agreed that the level of risk was substantially reduced following the application of the filters, and that rehousing was not necessary. Clear communication on the role and use of the filters was important to prevent their removal by residents, some of whom observed an impact on their water pressure. EHOs and the accommodation provider met with residents over the course of the outbreak to answer questions and address concerns.

As the outbreak progressed, a regimen of regular flushing (twice weekly) and disinfection (monthly) at the venue was established, including multiple shock disinfections with high-concentration chlorine solutions. However, post-disinfection samples continued to show high colonization levels, while biofilm breakdown following chemical treatment was felt to have led to more widespread colonization of the bacteria throughout the water system. Colonization levels continued to remain high even after the installation and use of a chlorine-based dosing unit. Consequently, the point-of-use filters were replaced every 3 months to provide an ongoing barrier to *Legionella* exposure.

Given the identified conditions favourable to *Legionella* growth, and the challenge of decolonizing the water system, the housing provider evaluated measures for structural changes. This included removing the ‘dead legs’ and insulating the piping. Following a review by contractors, challenges were identified in making the necessary changes. A significant proportion of the water pipes (>30%) were inaccessible without significant renovation work. Such work would have required the residents to vacate the premises and would have incurred significant costs for the housing provider. At the time of writing, renovation work is still outstanding, and the local authority remains engaged with the provider to ensure the work is completed prior to the removal of the filters. To reduce heat transfer, the housing provider has lowered the centrally set radiator temperatures overnight, allowing the accommodation to cool down.

The housing provider implemented the above control measures following an on-site inspection from the HSE at which a breach of the Health and Safety at Work, etc. Act 1974 was identified. The HSE enforcement action was related to management failings, including no recent review of the Legionella risk assessment, and the lack of a nominated and trained ‘responsible person’ on-site. The provider did engage with the HSE and had addressed the issues raised by Summer 2024.

## Conclusion and recommendations

We report an outbreak of LD at a private residence in Yorkshire, with two confirmed cases and one probable case identified. Unusually for an outbreak of LD, the most likely source here was exclusively the cold-water outlets at the residence.

While preventing *Legionella* colonization in a hot-water distribution system requires maintaining temperatures above 60°C [5], in cold-water systems, it is raised temperatures, often secondary to heat transfer, that predispose to bacterial growth [5]. In this outbreak, the structural pre-conditions favourable to bacterial growth included the presence of system ‘dead legs’ and inadequate pipe insulation in the context of persistently raised ambient air temperatures at the accommodation. While efforts to remove the system ‘dead legs’ were made, further structural changes to reduce heat transfer may have required the vacation of private tenants, at a significant cost to the provider. The IMT felt that engagement with the provider in implementing control measures improved markedly when HSE enforcement was actioned. There is some evidence in the literature to suggest that legislation and enforcement can improve public health outcomes [15]. While the initial application of the point-of-use filters occurred prior to HSE inspection, the IMT recognized that engagement around securing further improvements at the venue improved following formal HSE notification of the provider’s breaches. It is important to note that collaboration with regulators, such as the HSE and the local authority, may be necessary for action risk reduction measures, and when required, early regulator engagement at the IMT is important.

Finally, confusion arose in the investigation of this outbreak since the first case’s reported date of symptom onset occurred prior to their move to the residence. Consequently, on-site investigation did not occur until 34 days after the initial case notification, when the second case was identified and linked to the venue. This raises questions about the significance we assign to patient-reported dates of symptom onset when responding to health protection hazards. Early on, the question was raised whether this patient may have been experiencing symptoms of a prior, separate illness that overlapped with their LD. However, further investigation subsequently revealed that the case had a brief visit to the venue during the putative incubation period. We suspect he was exposed to *Legionella* from using the kitchen taps during this visit.

## Data Availability

The findings of this paper do not rely on any data, code, or other resources.
